# Temperature Monitoring Devices in Neonates

**DOI:** 10.3389/fped.2021.732810

**Published:** 2021-08-24

**Authors:** Donna Lei, Kenneth Tan, Atul Malhotra

**Affiliations:** ^1^Department of Paediatrics, Monash University, Clayton, VIC, Australia; ^2^Monash Newborn, Monash Children's Hospital, Monash Health, Clayton, VIC, Australia; ^3^The Ritchie Centre, Hudson Institute of Medical Research, Melbourne, VIC, Australia

**Keywords:** BEMPU, electronic, hypothermia, liquid crystal, mercury, neonate, thermometer

## Abstract

**Introduction:** Accurate temperature monitoring of neonates is vital due to the significant morbidities and mortality associated with neonatal hypothermia. Many studies have compared different thermometers in neonates, however, there is a lack of consensus regarding which of the currently available thermometers is most suitable for use in neonates.

**Objectives:** The aim of this review was to identify and compare current methods available for temperature monitoring of neonates beyond the delivery room, including the accuracy, advantages and disadvantages of each.

**Methods:** A recent search and narrative synthesis of relevant studies published between January 1, 1949 and May 5, 2021 on the OVID Medline, PubMed and Google Scholar databases.

**Results:** A total of 160 papers were retrieved for narrative synthesis. The main methods available for temperature monitoring in neonates are human touch and mercury-in-glass, electronic, infrared tympanic and other infrared thermometers. Newer innovations that are also available include liquid crystal thermometers and the BEMPU TempWatch. This paper discusses the current evidence available regarding the utility of these devices, and identifies barriers to valid comparison of different thermometry methods.

**Conclusion:** Many methods for temperature monitoring in neonates are currently available, each with their own advantages and disadvantages. However, the accuracies of different devices are hard to determine due to variable methodologies used in relevant studies and hence, further research that addresses these gaps is needed.

## Introduction

Neonatal hypothermia is a global problem that causes significant morbidity and mortality particularly in low- and middle-income countries. It is defined by the World Health Organization as an axillary temperature below 36.5°C (1) and is estimated to affect 11–92% of neonates (2). Risk factors include prematurity (birth prior to 37 weeks completed gestation) (3), low birth weight (birth weight <2500 g) (4, 5), low maternal socioeconomic status, younger maternal age (6) and birth outside of the hospital (7). Hypothermia during the neonatal period leads to significant short- and long-term complications. Within low- and middle-income countries, the main complication is mortality and studies have shown that hypothermia increases a neonate's risk of mortality by up to 23 times (8). Across all populations, neonatal hypothermia is also associated with morbidities including hypoglycaemia (9), jaundice, infections (7), respiratory distress syndrome, pulmonary hemorrhage (10) and intraventricular hemorrhage (11). Early detection of neonatal hypothermia allows for prompt mitigation and is therefore vital for reducing neonatal morbidity and mortality globally.

Substantial research has been conducted to determine the ideal method for temperature measurements in neonates. Consensus regarding the ideal method is that it should be simple, rapid, non-invasive, reproducible (12), cost-effective and accurately reflect the neonate's core body temperature (13). In this review, we discuss the different thermometer devices available for use in neonates beyond the delivery room, including commonly used thermometers as well as newer innovations. We compare the advantages and disadvantages of each, discuss methodological aspects of relevant studies, and suggest areas for further research to address gaps in the current literature.

## Search Methods

A search of the literature was performed to identify the current methods available for temperature monitoring in neonates. Relevant papers published between January 1, 1949 and May 5, 2021 were found by searching the OVID Medline, PubMed and Google Scholar databases, and limiting results to papers that focused on human newborns and were published in English. Table 1 shows the keywords used during this search. The reference lists of relevant articles were also cross-checked to identify further relevant studies. Studies that were eligible for inclusion were prospective research articles that occurred in settings beyond the delivery room, and which compared thermometry methods in neonates to provide an indication of the accuracy of the devices. Relevant articles from the three databases were imported into the Covidence systematic review software (Veritas Health Innovation, Melbourne, Australia) to remove duplicates and then screened according to the title and abstract to determine their relevance.

**Table 1:** Search strategy—keywords and Medline medical subject heading (MeSH) terms used.

**Concept**	**Search items**	**MeSH terms**
Temperature monitoring device	((temperature OR hypothermi^*^) AND (monitor^*^ OR measur^*^ OR detect^*^)) OR thermomet^*^	“body temperature,” “cold temperature,” “skin temperature,” “temperature” “hypothermia” “thermometers,” “thermometry”
Neonate	neonat^*^ OR newborn OR infant	“infant, newborn,” “neonatology,” “neonatal nursing” “infant,” “infant care”

* *Truncation*.

Relevant studies were then grouped by intervention type since different thermometry methods measure temperature through different mechanisms and are affected by different factors. The interventions were grouped under the following categories: human touch, mercury-in-glass thermometers, electronic thermometers, infrared tympanic thermometers, other infrared thermometers, liquid crystal thermometers and the BEMPU TempWatch. Where available, the sensitivity and specificity of different methods were used as a measure of thermometer accuracy. If these were not available, other measures of accuracy were used including mean temperature difference between methods and the Pearson correlation coefficient. A risk of bias assessment tool was not used, however relevant limitations to the validity of the studies were identified for inclusion in the review. The method of synthesis was decided a priori to be narrative, and hence results from the identified studies were then synthesized in a narrative review. Given that we could not include all relevant studies in the final review, included studies were selected based on larger sample sizes and lower risks of bias, and to ensure that the evidence regarding different thermometry methods could be adequately discussed. Inconsistencies between studies were analyzed and discussed throughout the review, and the advantages and disadvantages of different thermometry methods were identified for inclusion in a summary table (Table 2).

**Table 2:** Summary of current methods of temperature monitoring and their advantages and disadvantages.

**Temperature monitoring device**	**Advantages**	**Disadvantages**
Human touch	•Simple and quick •Inexpensive •Easy to implement	•Only accurate when performed by someone trained
Mercury-in-glass thermometer	•Traditionally considered gold standard	•Contains mercury •Long time required to reach stable temperature •Variable accuracy due to suboptimal use
Electronic thermometer	•Poses minimal risk •Provides rapid temperature readings •Probes allow for continuous monitoring and dual monitoring	•Variable accuracy depending on site •Skin measurements affected by environmental factors
Infrared tympanic thermometer	•Rapid and painless	•Variable accuracy depending on model used •Temperatures differ between protected & unprotected ears •Expensive
Temporal artery and mid-forehead infrared thermometers	•Rapid and painless •Causes minimal disturbance to the neonate	•Variable accuracy depending on site •Measurements affected by environmental factors •Expensive
Liquid crystal thermometry	•Simple and inexpensive •Able to be understood by non-literate and non-numerate carers •Accurate in detecting hypothermia <35.5°C	•Does not provide a specific temperature reading •Falls off occasionally
BEMPU TempWatch	•Continuous monitoring •Able to be understood by non-literate and non-numerate carers •Promotes skin-to-skin contact and weight gain	•Does not provide a specific temperature reading •Limited studies regarding its accuracy

## Results

We identified 5,889 papers using the search strategy described. After we excluded those that were not relevant to the research question, 160 remained.

**Figure 1:**
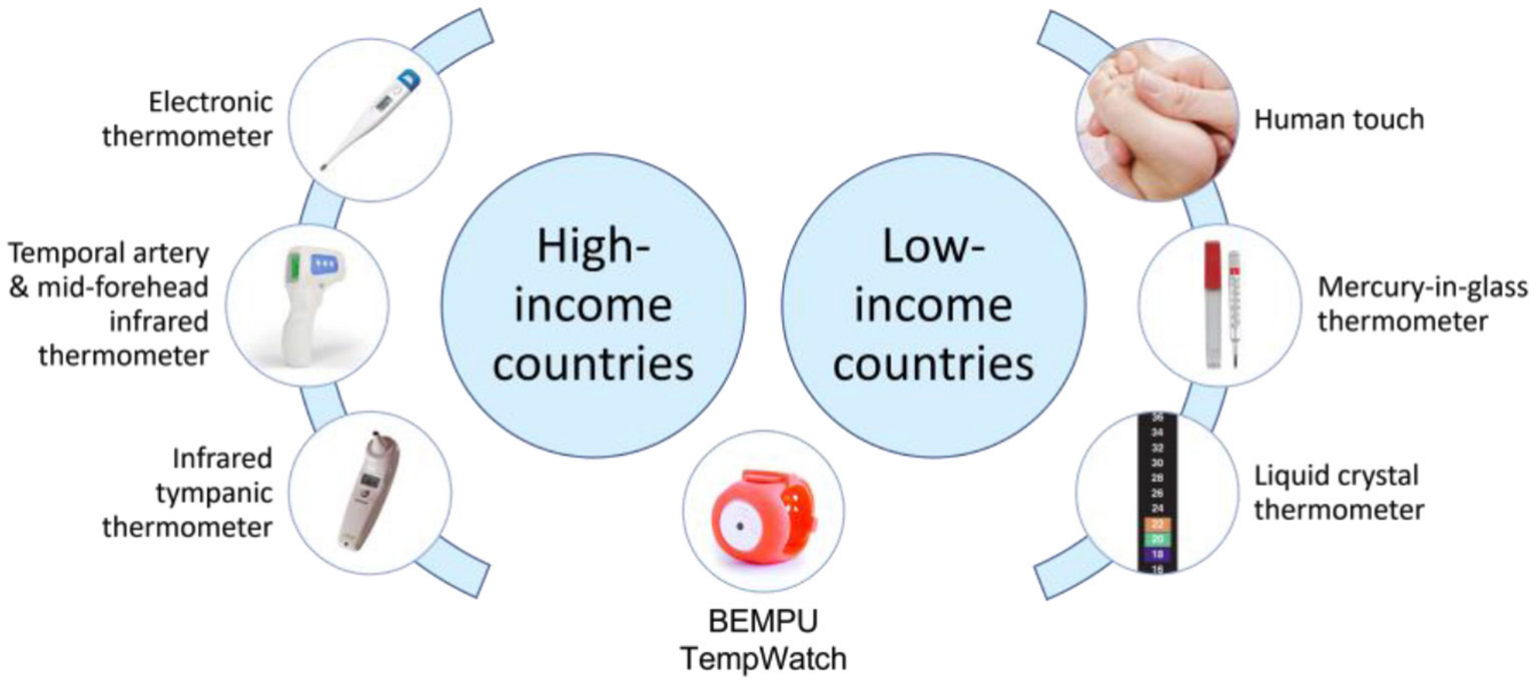
Temperature monitoring devices currently available for use in neonates.

Numerous different methods for temperature monitoring in neonates were identified and were shown to vary between high- and low-income countries (Figure 1). Their accuracies and relevant advantages and disadvantages are discussed below.

### Human Touch

The World Health Organization recommends human touch to detect hypothermia in resource-limited settings. This method involves simultaneous palpation of the baby at the abdomen and soles of their feet with the dorsum of the observer's hand (1). Warm abdomen and feet correspond to a warm baby (36.5–37.5°C), warm abdomen and cold feet to mild hypothermia (36–36.4°C) and cold abdomen and feet to moderate hypothermia (<36°C) (1). The benefits of human touch are that it is simple, quick, inexpensive and easy to implement (14). However, the accuracy of this method varies widely depending on who is conducting the measurements and whether they have been trained.

When conducted by untrained mothers, health workers and field workers, the accuracy of human touch to detect hypothermia is poor when compared to mercury-in-glass axillary thermometry. One study reported a sensitivity of 11–42% and specificity of 93–100% depending on the observer (15), whilst another found that only 24.6–34.4% of hypothermic babies were correctly identified (16). However, the accuracy of human touch improves when used to detect moderate hypothermia compared to mild hypothermia (15) and hence, it is recommended as a screening tool in a community setting where observations are made by untrained workers and mothers. Ellis et al. (15) emphasize the need for further palpation and monitoring if the baby is thought to be cold based on human touch. The accuracy of human touch increases when conducted by trained workers (14, 17) and pediatricians (18), with all pediatricians in one study found to be capable of correctly identifying all hypothermic babies.

### Mercury-in-Glass Thermometers

Mercury-in-glass thermometers have traditionally been considered the gold standard for temperature measurement and their use is still prevalent throughout low- and middle-income countries (19). However, they are no longer used in most high-income countries due to concerns about the risk posed by the mercury in them (20). Furthermore, when compared to newer electronic and infrared thermometers, mercury-in-glass thermometers take significantly longer to reach a stable temperature reading (21). Their accuracy is unclear as their recorded temperatures are often considered the gold standard against which other thermometers are compared (22, 23). Within clinical practice, their efficacy is often suboptimal due to uncertainties regarding where they should be placed and insufficient placement times (24).

The ideal location to place a mercury-in-glass thermometer in neonates is debatable, with rectal thermometry remaining the gold standard in many low- and middle-income countries (22). Whilst many have traditionally believed rectal temperatures to accurately reflect core temperature, numerous studies have now shown that rectal temperatures lag during rapid changes in core temperature (25) and are affected by the presence of feces and bowel organisms (26). The main advantage of placing mercury-in-glass thermometers in the rectum is the shorter placement time required for temperatures to stabilize. Kunnel et al. (24) found that 90% of rectal measurements reached their optimal temperature after 5 min, compared with 11 min for axillary measurements. However, rectal thermometry has been reported to cause significant complications in neonates. In multiple hospitals that experienced neonatal infection outbreaks, rectal thermometers were identified as the route of transmission (27), with organisms subsequently isolated from disinfected thermometers (28) and the disinfectant solutions in which the thermometers were stored (29). Rectal thermometers are also known to cause rectal perforations (30) when the probe is inserted too far or with too much force, leading to peritonitis, pneumoperitoneum (31) and mortality (32).

It is difficult to compare the accuracy of mercury-in-glass thermometers placed in the rectal and axillary sites due to a combination of reasons. Rectal temperatures vary depending on how deep into the rectum the probe is inserted and only reach a constant temperature at a depth of 5 cm (33). However, within studies comparing rectal and axillary thermometry, the depth of rectal probe insertion is often not stated (34, 35) or probes are only inserted to a depth of 2 to 3 cm (36, 37). Within these studies, the use of axillary thermometers is also often suboptimal (34) as they are kept in place for less than the 11 min required to reach stabilization (24). Two studies that compared these measurements after stabilization showed that axillary and rectal temperatures did not differ to a clinically significant degree of 0.2°C or more (38), with differences ranging from 0.02 to 0.1°C (12, 39). However, neither study inserted the rectal thermometer to a depth of 5 cm and hence comparison of the two sites based on existing studies remains difficult.

### Electronic Thermometers

Electronic thermometers are gradually replacing mercury-in-glass thermometers for routine monitoring especially in high-income countries. The advantages are that they pose minimal risk to the neonate, provide rapid temperature readings (40) and allow for continuous temperature monitoring when electronic probes are used (20). However, their accuracy is variable depending on which site measurements are taken from.

Although axillary and rectal sites both allow for continuous temperature monitoring in neonates, current evidence surrounding electronic thermometry supports axillary measurements whilst further research is needed regarding rectal measurements. When placed in the axilla, measurements by electronic thermometers have been shown to highly correlate to those made by traditional mercury-in-glass thermometers in preterm (36) and term (41, 42) neonates, with a mean difference of only 0.02°C (36). Indwelling rectal probes are commonly used for the continuous measurement of core temperature in neonates undergoing therapeutic hypothermia for the treatment of hypoxic-ischaemic encephalopathy (43). However, evidence regarding electronic rectal measurements in neonates is limited. Two studies which compared electronic and mercury-in-glass rectal thermometry found that many of the measurements differed by a clinically significant (38) 0.4 to 0.5°C (44, 45). However, in both studies rectal probes were only inserted 2 cm and mercury-in-glass thermometers were kept in place for 3 to 4 min rather than the 5-min optimal placement time needed (24). Hence, further studies with adequate insertion depths and placement times are required.

Electronic thermometers have also been designed for placement on the skin, with these probes commonly used for continuous temperature monitoring of neonates in incubators and under radiant warmers. Benefits of this site are that it poses minimal risk and allows for intermittent and continuous monitoring (20). The accuracy of skin measurements is highly variable however, as factors such as swaddling, clothing (46), the environmental temperature, how closely the thermometer is placed to the skin and the peripheral perfusion of the baby (20) affect the recorded temperature. Furthermore, skin temperatures have been shown to vary across the body, with higher temperatures over areas with large amounts of brown fat including the liver and intrascapular regions (47). Because different studies measure skin temperatures from different sites (48–50), the overall accuracy of electronic skin thermometry is difficult to determine. Early findings have shown that limiting placement of skin probes to areas of zero-heat-flux, such as between a neonate's skin and the mattress, allows recording of temperatures that reflect the neonate's core temperature (51) by creating an area of skin that is almost perfectly insulated (50).

Their ability to measure skin temperatures also means that electronic thermometers can continuously monitor two sites at once. Simultaneous monitoring of central and peripheral temperatures has been shown to provide valuable information on the health of newborns, particularly those that are premature or sick (20). Sites appropriate for the recording of central temperature include the abdomen or axilla, whilst the sole of the foot is typically used for peripheral measurements (52). Sustained changes in the central-peripheral difference in temperature are defined as a thermal gradient >2°C that is maintained for over 4 h (53) or which cannot be corrected with air temperature modifications (52), and have been shown to be an early indication of late-onset sepsis (53). Electronic probes can also measure nasopharyngeal (43), oesophageal (50, 54), bladder (55) and pulmonary artery (56) temperatures in neonates. However, although these sites are more representative of core temperature, their invasiveness often limits their use to neonates undergoing surgery (47) or, in the case of nasopharyngeal measurements, those who require nasogastric feeding tubes (57).

### Infrared Tympanic Thermometers

Infrared tympanic thermometers measure the infrared energy emitted from the tympanic membrane and surrounding tissue and convert it into a temperature reading through electronic thermal transducers (38). They are commonly used to measure the temperature of children and adults in high-income countries as they are rapid and painless (58) and have been shown to be accurate in these populations (59). Their use in neonates is currently limited and there is inconsistent information regarding their accuracy, which varies according to the population studied and the specific model used. Many different models of infrared tympanic thermometers are available for neonates, however only the FirstTemp Genius (model 3000A, Intelligent Medical Systems, Carlsbad, CA, USA) and Thermoscan (PRO-1 Instant Thermometer, Thermoscan, Inc., San Diego, CA, USA) models are discussed here as they have been the most widely researched.

The FirstTemp Genius infrared tympanic thermometer closely reflects axillary temperatures when used in sick neonates. Within a large-scale study conducted on sick newborns, temperatures measured by FirstTemp Genius and mercury-in-glass axillary thermometers differed by an average of 0.03°C (36), which did not reach clinical significance (38). Existing studies show that the accuracy of the FirstTemp Genius thermometer in healthy neonates, in either the rectal- or oral-equivalent modes, is low. In the rectal-equivalent mode, the FirstTemp Genius measured temperatures that were significantly higher than those measured by a mercury-in-glass rectal thermometer in both term (58) and preterm (12) neonates. No study compares the oral-equivalent mode to oral temperatures taken by another method of thermometry, most likely because oral temperatures require cooperation of the subject and are therefore difficult to obtain in neonates (60). However, when compared to mercury-in-glass rectal measurements, the FirstTemp Genius in the oral-equivalent mode was found to differ by 0.3°C or more for over 50% of measurements (58).

The Thermoscan infrared tympanic thermometer has been shown to accurately reflect core temperatures when used in a pediatric population aged 6 months to 15 years (61). The limited data regarding its accuracy in neonates have reported promising results. When compared to electronic axillary thermometry in term neonates, no clinically significant difference was found between temperatures measured by the two methods. One study found no statistically significant difference (62) whilst another reported a difference of 0.15°C (63), which still falls within the limits of clinical acceptability (38). In preterm neonates, the clinical utility of the Thermoscan thermometer is still unclear with Weiss (63) reporting that tympanic measurements were significantly higher than electronic axillary temperatures by 0.19 to 0.22°C. However, only 12 neonates were included within this study and hence larger-scale studies are needed to determine its accuracy in preterm babies.

Further research is needed to determine whether the accuracy of infrared tympanic thermometers varies across different clinical circumstances. Early studies have shown that tympanic temperatures in pediatric populations were not affected by postnatal age, the presence of vernix (13), otitis media (64), cerumen (65, 66) and fluid within the middle ear (67). However, the current literature does not address other clinical situations, including neonates undergoing certain treatments such as oxygen therapy or therapeutic hypothermia. Furthermore, within relevant studies, placement of neonates under radiant warmers or in incubators was shown to cause tympanic temperatures to be consistently higher than temperatures at other sites when compared to neonates nursed in open cots (46, 68). Another finding which must be considered when using infrared tympanic thermometers in neonates, was that the temperature measured differed depending on which ear was used. This finding was applicable to both the FirstTemp Genius (12) and Thermoscan (62, 63) models. All studies that reported this found that the ear that the neonate was lying on, also known as the protected ear, produced a higher temperature reading than the exposed ear (12, 63) by 0.2 to 0.3°C (62). It appears that using this ear may provide a better approximation of the rectal temperature (62) and allow closer estimation of the neonate's core body temperature.

### Infrared Thermometers at Other Sites

Infrared thermometers are also used at sites such as the temporal artery, mid-forehead, axilla (69) and leg (70). These thermometers measure the heat that radiates from the subcutaneous blood supply (71), allowing temperature measurement without direct contact with the baby. The main advantage is the minimal disturbance it causes the neonate. Studies comparing different characteristics before and after temperature measurement found that infrared thermometry led to less disturbance than axillary thermometry in terms of behavioral states (71), pain profiles, heart rate variation and partial oxygen saturation (19). The accuracy of temporal artery and mid-forehead measurements remains unclear and seems to vary depending on the site of measurement and the population studied.

The temporal artery is one of the main sites used for infrared thermometry as its connection to the heart via the carotid artery means it has a constant blood flow (72). Most studies regarding temporal artery thermometry compare it to electronic axillary thermometers and consistently report temporal artery readings to be higher. Haddad et al. (73) compared the two methods in healthy term and late preterm neonates and found that although temporal artery temperatures were higher than electronic axillary measurements, the difference was not clinically significant (38). However, when used in sick neonates in neonatal intensive care units, studies have shown that the difference between temporal artery and electronic axillary measurements exceeds the threshold for clinical significance (74). Sim et al. (75) analyzed this difference according to the neonate's environment and found that the difference between the measurements varied depending on the environment, ranging from 0.10°C for neonates in cots to 0.97 and 1.15°C for those under radiant warmers and in incubators, respectively. Furthermore, when compared to digital rectal thermometry, temporal artery thermometers were found to have a sensitivity of 73.6% and specificity of 52.9% in detecting hypothermia (<36.5°C).

Infrared mid-forehead thermometry has been proposed as an alternative to infrared temporal artery thermometry as the temporal artery area is small in neonates and hence difficult to use (19). Current studies show that mid-forehead measurements are inaccurate due to the various factors that influence the reading, including birthweight, the baby's environment (i.e., incubator or cot) and type of ventilator support used (76). Despite large study sizes, Uslu et al. (36) and Can et al. (77) found that mid-forehead thermometry correlated poorly to mercury-in-glass axillary thermometry (77). Restricting use of infrared mid-forehead thermometry to a standardized environment may make it a viable method of thermometry. When mid-forehead measurements were compared to electronic axillary thermometry in neonates nursed in incubators, temperatures measured by the two methods did not differ to a clinically significant degree (38).

### Liquid Crystal Thermometers

Liquid crystals selectively scatter light waves at specific wavelengths depending on the temperature (78). This is the basis of liquid crystal thermometry which changes color according to the baby's temperature. The most notable of these is ThermoSpot (Hallcrest, Glenview, IL, USA), a reusable plastic disc that sticks onto the baby's skin and changes from green to black when their temperature falls below 35.5°C (79). It can remain on for seven to 10 days and is marketed as a cost-effective hypothermia indicator that can be used by non-literate and non-numerate carers (79).

Within hospital studies, the reported accuracy of ThermoSpot differs due to variations in the definition of hypothermia used, which ThermoSpot defines as <35.5°C (79). Pejaver et al. (23) compared ThermoSpot to mercury-in-glass rectal thermometry in neonates using the ThermoSpot definition of hypothermia and found that ThermoSpot agreed with rectal temperatures 99.04% of the time. This was applicable across normothermic and hypothermic temperatures, with ThermoSpot correctly identifying every case of hypothermia. Kambarami et al. (80) reported that ThermoSpot had an overall accuracy of 57% and a sensitivity of 19% in detecting hypothermia, however they defined hypothermia as <36°C. Comparing studies regarding ThermoSpot is made even more difficult as the site of disc placement differs, with some placing it in the supraclavicular region while others place it over the liver area and axilla. Since it is known that skin temperatures vary across different parts of the body (47), this variation makes it difficult to compare relevant studies.

ThermoSpot is also a feasible and affordable temperature device for low-resource community settings. Green et al. (81) compared ThermoSpot to electronic axillary thermometry in babies that were born at home in Indian slum dwellings. Although they defined hypothermia as a temperature <35°C, ThermoSpot had a sensitivity of 88% and specificity of 97% in detecting hypothermia. The ThermoSpot device has not been reported to cause any skin damage or discomfort (81) and although it did occasionally fall off babies during the studies, it was easily reapplied with tape (23).

### BEMPU TempWatch

The BEMPU TempWatch (BEMPU Health, Bangalore, Karnataka, India) is a novel bracelet device that allows continuous temperature monitoring for 30 days (82). It consists of a thermistor metal cup within a plastic casing and a silicone band that is worn around the wrist of neonates who weigh between 800 to 3,300 g (83). The TempWatch indicates to carers when the neonate is hypothermic (<36.5°C) through an audio-visual alarm, at which point parents are encouraged to provide skin-to-skin contact to their babies (82).

Currently only one study has been conducted to determine the accuracy of the TempWatch in detecting hypothermia. Tanigasalam et al. (84) compared the TempWatch to mercury-in-glass axillary thermometry and reported it to have a sensitivity of 98.6% and specificity of 95% in detecting hypothermia. However, only neonates weighing <2,000 g were included in this study and the TempWatch was only worn for 24 h. Therefore, further studies are needed to determine whether this accuracy is applicable to neonates weighing 2,000 g or more and if the accuracy is maintained throughout the entire 30 days that the device is marketed for (82). Using the TempWatch for 4 weeks has been shown to promote parental compliance to skin-to-skin contact and neonatal weight gain during the first and fourth weeks of use (85). Across hospital and community settings, the device has also been shown to be accepted well by doctors and families (86), and has not been reported to cause any adverse effects to the neonate (85, 87). Since the TempWatch is a relatively new device, further research is needed to validate these early findings.

### Other Methods

The advantages and disadvantages of the thermometry methods identified are listed in Table 2.

Other methods for temperature monitoring in neonates include the chemical dot and pacifier thermometers. Currently, limited studies have been conducted to determine the utility of these two methods.

The chemical dot thermometer is designed for use at the oral, axillary or rectal sites and is a flexible polystyrene plastic strip that consists of a matrix of 50 dots, each containing a specific chemical mixture that changes color from beige to blue (88) according to their melting point (47). Each dot represents an increment of 0.1°C and, after stabilization, is able to be read according to the last dot that changed to blue (88). The chemical dot thermometer can detect temperatures ranging from 35.5 to 40.4°C, with axillary temperatures generally available after a stabilization period of 3 min (88). A disadvantage of this method, however, is that instruction is required prior to use (89).

The pacifier thermometer measures supralingual temperatures (90) and consists of either temperature-sensitive crystals or a thermistor placed inside the nipple of a pacifier with a digital display at the front on which the temperature is displayed (47). Within studies that compared pacifier thermometers to other methods, no subgroup analysis of neonates was conducted (91, 92). However, results of these studies showed that many parents did not want their infants using pacifiers (90) and many infants were unable to suck on the pacifier long enough to allow a steady temperature reading (91). Although pacifier thermometers are easy to understand, these findings limit their utility in neonates.

## Conclusions

The ideal method of temperature measurement should be simple, rapid, non-invasive (12), accurate and cost-effective (13). Numerous methods have been identified for use in neonates, with newer methods including electronic and infrared thermometers widely used in high-income countries whilst the traditional mercury-in-glass thermometer remains the gold standard within low-income countries. Newer innovations, including ThermoSpot and BEMPU TempWatch, aim to provide an accurate thermometry method that can be used in low- and middle-income countries. Due to the lack of consensus regarding a gold standard method of temperature measurement, as well as suboptimal methodologies used within studies, the accuracies of different thermometers are difficult to determine based on the current research available. Given the variation in temperature across the body, further research in this area should determine how temperatures at different sites vary from core temperature and focus on comparing different thermometers at the same site to allow for valid comparison of thermometry methods.

## Author Contributions

AM: concept. DL: data collection, compilation, and first draft of the manuscript. DL, KT, and AM: design, editing, and final approval of manuscript. All authors contributed to the article and approved the submitted version.

## Conflict of Interest

The authors declare that the research was conducted in the absence of any commercial or financial relationships that could be construed as a potential conflict of interest.

## Publisher's Note

All claims expressed in this article are solely those of the authors and do not necessarily represent those of their affiliated organizations, or those of the publisher, the editors and the reviewers. Any product that may be evaluated in this article, or claim that may be made by its manufacturer, is not guaranteed or endorsed by the publisher.
